# Review Article: Spectrum of Biliary Infections in the West and in the East

**DOI:** 10.1155/1995/74384

**Published:** 1995

**Authors:** H. G. Beger, A. Schwarz

**Affiliations:** 1 Department of General Surgery University of Ulm Germany; 2 Department of General Surgery University of Ulm Steinhövelstr. 9 Ulm 89075 Germany

## Abstract

Biliary infections are an important cause of morbidity in the Western world. With regard to epidemiology, etiology, microbiological spectrum, prevalence, location and composition of gallstones, pathogenesis, clinical sign and therapy, there are large differences between the spectrum of biliary infections in the East and in the West (Table 1). In Western countries, gallstones are found in 10 to 40%. In Eastern countries, the incidence of gallstones is only 2 to 6%. Some eighty – five percent of the gallstones in the West are cholesterol stones, in contrast to the East, where 97% are bile pigment stones. The most important difference is characterized by the origin of common bile duct stones. In the West, common bile duct stones generally originate in the gallbladder, in contrast to the East, where primary common bile duct stones are often found – especially in the intrahepatic segments – with no evidence of gallbladder stones. The sex distribution male to female in the West is 1:2, in the East 1:1. In the West, biliary infections occur mainly in an elderly population, 50% being older than 70 years. In the East, biliary infections appear also in younger people, 50% being younger than 40 years. Parasites play an aetiological role in the East, but not in the West. The typical therapy of gallstones in the West is cholecystectomy, and of common bile duct stones endoscopic sphincterotomy. Due to the frequency of intrahepatic stones in Eastern Countries, the therapeutic spectrum there includes even large hepatic resections and biliary enteric anastomoses.


*HPB Surgery*, 1995, Vol. 8, pp. 215–222
Reprints available directly from the publisher
Photocopying permitted by license only

© 1995 OPA (Overseas Publishers Association)
Amsterdam B.V. Published in The Netherlands
by Harwood Academic Publishers GmbH
Printed in Malaysia

### Review Article: Spectrum of Biliary Infections in the West and in the East

H. G. BEGER* and A. SCHWARZ

Department of General Surgery, University of Ulm, Germany

*(Received April 14, 1994)*

Biliary infections are an important cause of morbidity in the Western world. With regard to epidemiology, etiology, microbiological spectrum, prevalence, location and composition of gallstones, pathogenesis, clinical sign and therapy, there are large differences between the spectrum of biliary infections in the East and in the West (Table 1). In Western countries, gallstones are found in 10 to 40%. In Eastern countries, the incidence of gallstones is only 2 to 6%. Some eighty–five percent of the gallstones in the West are cholesterol stones, in contrast to the East, where 97% are bile pigment stones. The most important difference is characterized by the origin of common bile duct stones. In the West, common bile duct stones generally originate in the gallbladder, in contrast to the East, where primary common bile duct stones are often found – especially in the intrahepatic segments – with no evidence of gallbladder stones. The sex distribution male to female in the West is 1:2, in the East 1:1. In the West, biliary infections occur mainly in an elderly population, 50% being older than 70 years. In the East, biliary infections appear also in younger people, 50% being younger than 40 years. Parasites play an aetiological role in the East, but not in the West. The typical therapy of gallstones in the West is cholecystectomy, and of common bile duct stones endoscopic sphincterotomy. Due to the frequency of intrahepatic stones in Eastern Countries, the therapeutic spectrum there includes even large hepatic resections and biliary enteric anastomoses.

KEY WORDS: Biliary infection gallstones epidemiology bacteriology pathogenesis

### INTRODUCTION

Operations on the biliary tree are more frequent than any other major surgical intervention in the abdomen. Under normal conditions human bile is sterile. Under pathological conditions bacteria can invade the biliary tract through directly ascending infection from the duodenom or hematogenously from the hepatic portal venous blood.

### PREVALENCE, COMPOSITION AND ORIGIN OF GALLSTONES

Epidemiological studies show a large difference of the prevalence of gallstones in different racial groups (Table 2). In the West, there is a high incidence of gallbladder stones, ranging from 10 to 40% (Table 2) and showing large racial differences[1–3]. The highest prevalences of gallstones are reported from Chile (35%), Sweden (36%), from American Indians (30%) and from whites in North America (24%). Certain American Indian communities have some of the highest prevalence rates in the world which is due to the production of supersaturated bile. Sampliner[4] found the overall prevalence in Pima Indian females to be 48,6%. In Europe, the prevalence is 15 to 20%[1]. Godfrey[5] correlated autopsy and cholecystectomy data and observed that the prevalence of gallstones in the adult English population is 17%. The sex distribution male to female is approximately 1:2. About seventy-five percent of the stones are cholesterol stones (6). In patients with cholesterol stones, the bile is infected in 30–40% of cases[6,7]. Cholesterol stones are thought to originate in the gallbladder[8]; they are probably of metabolic origin.

*Correspondence and reprint requests to: Prof. H. G. Beger, Department of General Surgery, University of Ulm, Steinhövelstr. 9, 89075 Ulm, Germany

In the East, the prevalence of gallstones is low, ranging from 2 to 6%[1]. The sex ratio male to female is approximately 1:1[6], about 75% of the stones being bile pigment stones. Bacteria are said to play a major role in the formation of bile pigment stones[9]. In cases of bile pigment stones, the bile is infected in 95–100%[7]. In the Hong Kong Chinese population, 75% of the gallstones are found in the common bile duct. In Japan, the situation was found to be just between Europe and China. Gallstones are found in Japan in about equal frequency in gallbladder and bile duct[6]. The composition of gallstones in Japan is gradually changing from once predominant bile pigment stones to cholesterol stones, thus approaching that of the West. The percentage of cholesterol stones in Japan increased from 34% in 1938 to 85% in 1968[10]. The decreasing prevalence of bilirubin stones in Japan may be related to multiple factors including eradication of parasites and westernization of the diet[11,12].

In the West, common bile duct stones usually have their origin in the gallbladder. Intrahepatic stones are extremely rare in the United States and in Europe[8,13]. In the Far Eastern countries, including China, Japan, Taiwan[14] and Hong Kong, cholangitis is often associated with primary common bile duct stones, especially in the intrahepatic segments, with no evidence of gallstones or gallbladder disease[15]. Among patients with gallstones in the East, the prevalence of intrahepatic stones ranges from 3 to 10%[11,16,17], 88% of intrahepatic stones being bile pigment stones[11]. Patients with intrahepatic cholelithiasis are young. Onset of symptoms in over a third of patients occurs before 20 years of age. The sex distribution male to female is 1:1. The Southeast Asian intrahepatic stones are multiple, soft, muddy, pigmented, often adherent to the ductal wall, and usually located in the primary and secondary biliary radicals of the left liver lobe[14]. Although the cause of primary intrahepatic stones is unknown, a congenital cystic dilatation of the common bile duct associated with biliary strictures is often found. These strictures usually occur in the hilum or in some parts of the intrahepatic biliary tract, the stones being impacted in the bile duct proximal to the stricture. These strictures seem to be a congenital dysplasia of the biliary ductal system[15]. For treatment of these mostly young patients, a radical operation with hepatic resection including lateral segmentectomy, left hepatic lobectomy, or anterior segmentectomy, and biliodigestive anastomosis is often necessary[15]. The increased prevalence of intrahepatic stones in Japanese patients compared with the West is due to the increased prevalence of bilirubin stones. The cause of formation of these stones remains unknown, but they are thought to be related to stasis and infection. The confirmed presence of bile pigment stones in Japan, despite virtual eradication of parasites after 1965 and the continued urban–rural differences in the frequency of intrahepatic stones, suggest that one factor could be the rural diet, low in saturated fat and protein[11], which causes cholecystokinin release and sphincter of Oddi relaxation. The high prevalence of intrahepatic stones in Brazil[18], a rural country genetically unrelated to Japan, supports this theory. This rural low–protein diet may increase the quantity of unconjugated bilirubin by decreasing glucuronolactone, a major inhibitor of bacterial $\beta$-glucuronidase[19].

### CLINICAL SPECTRUM, PATHOGENESIS AND INCIDENCE OF BILIARY INFECTIONS

### *Cholecystitis*

The most common form of acute cholecystitis is acute obstructive (calculous) cholecystitis. It accounts for 90-95% of cases. It results from cystic duct obstruction by a stone. The gallbladder becomes acutely inflamed with transmural oedema. The current consensus is that the initial inflammation is chemically induced and not of bacterial origin. The currently held hypothesis is that mucosal trauma releases phospholipase which converts the lecithin in the gallbladder bile to lysolecithin, a mucosal toxin.

The acute acalculous cholecystitis accounts for up to 8% of acute cholecystitis[20] and was observed to increase in frequency in the last 30 years[21]. It develops on a background of prolonged illness, e.g. multiple trauma, severe sepsis or major surgery[22]. The aetiology remains unknown. The presumed pathophysiology involves gallbladder distension, bile stasis together with a mucosal injury and vascular occlusion, leading to acute inflammation of the gallbladder.

The most important cause of chronic cholecystitis are gallstones. Some sixty–five to seventy percent of patients with symptomatic gallstones develop chronic cholecystitis[23].

### *Cholangitis*

The essential elements in the development of cholangitis are bile stasis, foreign bodies (i.e. stones) and bacterial colonization.

Bacterial colonization of the common bile duct can result from duodenal reflux or from portal bacteraemia. Partial or total obstruction leads to bile stasis, setting the stage for bacterial proliferation, inflammatory response of the bile duct and cholangitis. We have to distinguish 5 different types of chlogangitis. The ascending or nonsuppurative cholangitis is caused by a partial obstruction of the biliary tree. It is the most common type of this disease today. Suppuration does not occur, because the infected contents are not under pressure.

In contrast, suppurative obstructive cholangitis, which accounts for 10–15% of cases, is associated with total or near-total obstruction of the biliary system. In this setting, inflammatory cells and bacteria accumulate rapidly within the bile duct and, because of the pressure, bacterial hepatitis, sepsis, multiple organ failure and ultimately death will occour if the bile duct is not decompressed. In Western countries, half of the patients with cholangitis are older than 70 and it is unusual under 50[24].

The most common aetiological factors of cholangitis in the Western countries (Table 1) are stones formed in the gallbladder (80% of cases), bile duct obstruction due to benign or malignant stricture and biliary tract operations with biliary enteric anastomoses, i.e. choledochoduodenostomy, choledochojejunostomy or sphinteroplasty[25]. Cholangitis can also appear after biliary instrumentation, such as ERCP[26], T-tube cholangiography[27] and transhepatic cholangiography.

The Oriental cholangiohepatitis (recurrent pyogenic cholangitis) is an endemic disease in Southeast Asia[28–33]. It is common among the younger Asian population and is characterized by recurrent attacks of abdominal pain, fever and jaundice. Pathologically, the intra- and extrahepatic ducts are dilated and contain soft, pigmented stones and pus. Localized intrahepatic segmental ductal stenosis may be present, especially in the lateral segment of the left lobe or posterior segment of the right hepatic lobe. The cause of the disease is not known. Some authors suggest a primary infection of the intrahepatic bile ducts with bacteria from the intestine[34], others think of dietary factors like low-protein diet[31].

**Table 1** Spectrum of biliary infections:Essential differences between West and East (1, 6–9, 12–15, 24–26, 28, 30, 33–41, 43, 70)

| | *WEST* | *EAST* |
|---|---|---|
| Incidence of GB stones | 10–40% | 2–6% |
| Incidence of GB stones among patients with gallstones | 99–100% | 20–40% |
| Incidence of intrahepatic stones among patients with gallstones | <1% | 3–10% |
| Origin of CBD stones | 2% primary stones, 98% secondary stones originating in the GB | >80% primary CBD stones |
| Composition of gallstones: percentage of cholesterol stones | 85% | 3% |
| Composition of gallstones: percentage of bile pigment stones | 15% | 97% |
| Age of patients with biliary infections | elderly population 50% > 70 years | younger population 50% < 40 years |
| Sex distribution | male:female = 1:2 | male:female = 1:1 |
| Aetiological factors of biliary infections | GB stones (80%), bile duct obstruction, endoscopic sphincterotomy, biliary enteric anastomosis | parasites like clonorchiasis sinensis, ascariasis, opisthorchis viverrini in 40% of patients with recurrent pyogenic cholangitis |
| Microbiological spectrum | aerobes common (87–94%), anaerobes uncommon (6–30%) | anaerobes more frequent |
| Typical therapy | cholecystectomy, endoscopic sphincterotomy | sometimes hepatic resection, biliary enteric anastomosis |

GB = gallbladder; CBD = common bile duct.

**Table 2** Distribution of gallstones in the West and in the East (1–5)

| *Continent* | *Country* | *Author* | *male* (%) | *female* (%) | *total* (%) |
|---|---|---|---|---|---|
| Europe | Sweden | Lindström | 26.5 | 46.8 | 36.2 |
| | Norway | Torvik | 13.5 | 28.6 | 20.1 |
| | Germany | Rodewald | 8.9 | 29.8 | 15.8 |
| | GB | Bouchier | 9.5 | 19.2 | 15.5 |
| America | North America (whites) | Newman | 16 | 32.5 | 24.3 |
| | Panama (whites) | Hall | 10.9 | 22.2 | 16.6 |
| | Panama (negroes) | Hall | 6.4 | 10.8 | 8.6 |
| | American Indians | Reichenbach | 16.6 | 40 | 29.9 |
| | Chile | Marinovic | 20.5 | 50 | 35.2 |
| Africa | Zaire | Trowell | | | 0.7 |
| | South Africa (whites) | Becker | 10 | 19.5 | 14.7 |
| | South Africa (Bantus) | Becker | 1 | 3.8 | 2.4 |
| Australia | | Joske | 11.3 | 20.3 | 14.9 |
| Asia | Japan | Maki | | | 4.4 |
| | Singapore | Hwang | 6.4 | 7.9 | 6.6 |
| | Korea | Hur | | | 4.5 |
| | Thailand | Stitnimankarn | 1.8 | 3.9 | 2.9 |

Association with parasites like clonorchiasis sinensis, ascariasis or opisthorchis viverrini has also been suggested, however [33,35,36]. Stool examination for parasites revealed clonorchis sinensis in 11 to 38% of patients with recurrent pyogenic cholangitis[28,37,38]. Oriental cholangiohepatitis is associated with primary common bile duct stones which are usually pigmented bilirubinate stones. Typically, they are located in the intrahepatic segments, with no evidence of gallstones or gallbladder disease[34,39]. Pathologically, the intra- and extrahepatic ducts are dilated and contain soft, pigmented stones and pus. Localized intrahepatic segmental ductal stenosis may be present, especially in the lateral segment of the left lobe or posterior segment of the right hepatic lobe.

Biliary strictures can be found in 35% of cases. Gallbladder stones are relatively uncommon (20–40%) in comparison with Western-type biliary disease[38,40,41].

Only sporadic cases of recurrent pyogenic cholangitis have been reported in Europe[42] and South Africa[43]. But with increasing migration of people from Eastern to Western countries, its incidence, especially in the United States and Canada, is rising[31,32,34]. In contrast to gallstone disease in Western countries, recurrent pyogenic cholangitis affects a younger age group, 50% being younger than 40[28], and the sex ratio male to female being 1:1 (Table 1).

Cryptogenic cholangitis is a microscopic form of the disease that affects the intrahepatic portion of the biliary system[45]. We do not know its cause, but it has been related to and found in systematic diseases, including the toxic shock syndrome[46].

Primary sclerosing cholangitis is a progressive cholestatic disorder characterized by a fibrosing inflammatory process which affects the intra- and/or extrahepatic ducts. Approximately seventy percent of patients with sclerosing cholangitis are men and 70% of patients are younger than 45 years[47]. The aetiology has not yet been defined. Infection and immunological factors have been discussed. Patients with sclerosing cholangitis have a high incidence of ulcerative colitis ranging between 25 and 74%[48,49]. Warren[50] reported that E. coli has grown in many of his patients. He pointed to the association with ulcerative colitis in which the mucosal barrier is interrupted, facilitating the entrance of bacteria into the portal circulation and the evolution of an inflammatory sklerosing process in the ducts. Chapman[51] observed a 60% incidence in the frequency of HLA-B8 in patients with sclerosing cholangitis, compared with 25% incidence in controls. These results suggest an immune aetiology of sclerosing cholangitis.

### BACTERIOLOGICAL SPECTRUM

### *Incidence of bacteria in the biliary tract*

The bile in persons without any biliary tract disease is sterile[52–55]. The incidence of positive bile cultures in patients with gallbladder stones (Table 3) varies in

**Table 3a** Bacteriology of the bile: number of positive cultures (7,12, 52–61,63–65)

| *Patients* | *Bile from GB/CBD* | *Number of positive cultures* |
|---|---|---|
| Normal GB, no stones | GB | 0% |
| Stones in the GB | GB | 8–30% |
| Stones in the CBD | CBD | 51–88% |
| Cholesterol stones | CBD | 33% |
| Bile pigment stones | CBD | 100% |
| Patients after EST | CBD | 70–75% |
| Biliary enteric anastomosis | CBD | 70–90% |
| Malignant bile duct obstruction | CBD | 10–30% |
| Acute cholecystitis | GB | 80% |

GB = gallbladder; CBD = common bile duct; EST = endoscopic sphincterotomy.

**Table 3b** Bacteriology of the bile:frequency and type of bacteria

| | | | |
|---|---|---|---|
| Aerobic | gram positive | Streptococci | 11–46% |
| | gram negative | E. coli | 17–52% |
| | | Klebsiella | 3–21% |
| | | Enterobacter | 3–9% |
| | | Pseudomonas | 2–15% |
| | | Proteus | 2–8% |
| Anaerobic | gram positive | Clostridia | 6–13% |
| | | anaerobic Cocci | 3–8% |
| | gram negative | Bacteroides | 3–8% |

different reports between 8 and 30%[52–60]. Examination of gallbladder wall in patients with gallbladder stones revealed an incidence of positive cultures between 25 and 50%[53,61,62]. In patients with choledocholithiasis, the incidence of infected bile is significantly higher, ranging from 51 to 88%[12,53,54,56,61,63–65]. Bacteria are more common in bile if the patient is jaundiced, and particularly if biliary obstruction is due to stones or a bile duct stricture. In acute cholecystitis, positive cultures were observed in 60 to 80% of cases[56,58,59]. In patients with benign bile duct strictures and in patients with a previous biliary enteric anastomosis, the incidence of bacterial proliferation in the biliary tract varies between 70 and 90%[56,57]. In patients with malignant obstruction the number of bacteria in the bile is significantly lower, ranging from 0 to 30%[56,62,63]. After endoscopic sphincterotomy, infected bile was found in about 70% of patients[66,67]. If the lumen of the gallbladder is infected, the bile duct is always colonized by the same bacteria. In patients with bacteria in the gallbladder bile organisms can be isolated from 80% of liver biopsies, from 80% of duodenal aspirates, from the wall of the gallbladder in 88%, and from the cystic lymph node in 60% of cases[56]. Conversely, if the gallbladder bile is sterile, all the other sites tested are sterile as well, except the duodenum (52% infected) and the gallbladder wall (30% infected).

### *Spectrum of bacteria in infected bile*

The bacteria counts in infected bile vary up to approximately $10^9$ organisms per ml. Patients with complete extrahepatic biliary obstruction occasionally show extremely low bacteria counts ($< 10^2$ organisms/ml). The predominant organisms in infected bile are aerobic bacteria (70–90%), particularly Escherichia coli (26–52%), Klebsiella species (15–21%) and Streptococcus faecalis (13–46%), which belong to the intestinal microflora[53,56,58,59,63]. Anaerobes can be seen only in about 10 to 30%, particularly Bacteroides species and Clostridium species. Bacteroides fragilis is commonly recovered after a previous biliary-enteric anastomosis, particularly in the presence of a recurrent stricture[68]. The predominant species after endoscopic sphincterotomy are of gastrointestinal origin:E. Coli, Enterococcus, Proteus, Klebsiella[66,69] and Pseudomonas[67]. In the bile of patients with recurrent Oriental cholangiohepatitis anaerobes are more common.

Bile is usually colonized by more than one organism in 62%. In patients with positive bile culture Keighley[56] isolated a single bacterial species in only 38%.

Two species were identified in 29%, three species in 20% and four species occurred together in 12%.

### SOURCE OF BACTERIA

The entry of bacteria into the bile remains controversial. It appears that bacteria may pass from the gastrointestinal into the biliary tract either by an ascending route or by the hematogenous route via the portal blood.

The most probable route of infection is the directly ascending spread from the duodenum into the common bile duct and the gallbladder[63,70,71]. Positive bile cultures are most commonly found in patients with ductal stones or carcinoma of the ampulla, which produce intermittent obstruction of the bile flow. Another increasingly common mechanism for bacterial ascent results from disruption of the sphincter of Oddi following endoscopic sphincterotomy[66,71–74]. The third mechanism for bacterial reflux results from biliary tract operations with biliary enteric anastomosis.

The other potential route of bacterial invasion into the biliary tract is via the portal venous blood[75,76]. Blood throughout the human portal venous system without any gastrointestinal infection is normally free from bacteria[77]. In typhoid carriers, Salmonella typhi harbored in the gallbladder enter the biliary tract as a consequence of portovenous bacteraemia. In a study of portal blood cultures Schatten[78] obtained positive cultures in 32% of patients undergoing upper abdominal surgery. Experimental infusion of bacteria into the portal circulation results in these bacteria appearing in bile[75]. These findings suggest that there is also a periodic delivery of bacteria from the intestine into the portal circulation. These enteric organisms are either routinely filtered by the liver or excreted in the bile. If common bile duct is obstructed, infection can ensue.

### PHYSIOLOGICAL DEFENSE MECHANISMS PREVENTING BILIARY INFECTION

To guard against bacterial invasion from duodenal reflux and from portal venous bacteraemia, there exist several mechanical, physical, chemical and immunological defense mechanisms.

### *Mechanical barriers*

The sphincter of Oddi, which separates the colonized duodenum from the uncolonized biliary tract acts as a mechanical barrier to microbial colonization[71].

Tight junctions between hepatocytes seal the bile canaliculus from the sinusoidal blood. The integrity of the tight junctions between hepatocytes is important in preventing the entry of bacteria from the sinusoidal blood into the biliary tract.

### *Physical mechanisms*

Throughout the day an average of 800–1000 ml of bile flushes the bile duct. The physical movement of bile hinders bacteria from colonizing the biliary tract. In biliary obstruction, bile flow and bile salt secretion decline[79]. The reduction of the flushing effects of bile might predispose patients to biliary infection.

### *Chemical factors*

Bile salts have an inhibitory effect on the proliferation of enteric micro-organisms and thus might help to prevent biliary tract infections. Both conjugated and unconjugated bile salts, at physiological concentrations, have been shown to inhibit E. coli, Klebsiella species and Enterococcus species in vitro[80].

### *Immunological defense*

Kupffer cells play a complex role in the defense against biliary infection[81]. They are strategically located inside the liver sinusoids to filter endotoxins and bacteria that come in through the portal circulation by phagocytosis.

The predominant immmunoglobulin in bile is IgA. Secretory IgA can bind and thus prevent bacteria from attaching to and penetrating intestinal epithelial cells[82]. In patients suffering from cholestasis due to biliary obstruction, the excretion of IgA through the liver is impaired. The antibacterial adherence effect of secretory IgA might play an important role in avoiding microbial colonization of the biliary tract.

### SUMMARY

In this article, the literature is reviewed to show the spectrum of biliary infections in the West and in the East. With regard to epidemiology, etiology, prevalence, location, composition and origin of gallstones, microbiological spectrum, pathogenesis, clinical signs and therapy, large differences in the spectrum of biliary infections in the East and in the West can be seen. In the West, there is a high incidence of gallbladder stones, ranging from 10 to 40%. In the East, gallstones were found in only 2 to 6%. Among patients with gallstones, intrahepatic stones were found in 3 to 10% in the East, but in less than 1% in the West. In the West, common bile duct stones generally originate in the gallbladder, in contrast to the East, where more than 80% of common bile duct stones are primary common bile duct stones. Some eighty-five percent of stones in the West are cholesterol stones. In Eastern countries, 97% of stones are bile pigment stones. In the West, the most common therapy of gallstones is cholecystectomy, and in case of common bile duct stones endoscopic sphincterotomy. Due to the frequency of intrahepatic stones in Eastern countries, the therapeutic spectrum there includes even large hepatic resections and biliary enteric anastomoses.

### REFERENCES

1. Brett, M. and Barker, D. J. P. (1976) The world distribution of gallstones. *Int. J. Epidem.*, **5**, 335–341.
2. Eisenburg, J. (1983) Akute und chronische Gallenwegsinfektionen. *Fortschritte der Medizin*, **101**, 1407–1412.
3. Bouchier, I. A. D. (1988) Gallstones:formation and epidemiology. In *Surg. liv. bil. tract*, edited by L.H. Blumgart, **1**, 503:516. Churchill Livingstone.
4. Sampliner, R. E., Bennett, P. H., Commess, L. J., Rose, F. A. and Burch, T. A. (1970) Gallbladder disease in Pima Indians. Demonstration of high prevalence and early onset by cholecystography. *N. Eng. J. Med.*, **283**, 1358.
5. Godfrey, B. J., Bates, T., Harrison, M., King, M. B. and Padley, N. R. (1984) Gallstones and morbidity. A study of all gallstone-related deaths in a single health district. *Gut*, **25**, 1029.
6. Miyake, H. and Johnston, C. G. (1968) Gallstones: Ethnological studies. *Digestion*, **1**, 219-228.
7. Stewart, L., Smith, A. L., Pellegrini, C. A., Motson, R. W. and Way L. W. (1987) Pigment gallstones form as a composite of bacteriological microcolonies and pigment solids. *Ann. Surg.*, **206(3)**, 242–249.
8. Glenn, F. and Moody, F. G. (1961) Intrahepatic calculi, *Ann. Surg.*, **153**, 711–734.
9. Maki, T. (1966) Pathogenesis of calcium bilirubinate gallstones: Role of $\beta$-glucuronidase and coagulation by inorganic ion, polyelectrolytes and agitation. *Ann. Surg.*, **164**, 90–100.
10. Nakayama, F. and Miyake, H. (1970) Changing state of gallstone disease in Japan. Composition of gallstones and treatment of the condition. *Am. J. Surg.*, **120**, 794–799.
11. Nagase, M., Hikase, Y., Soloway, R. D., Tanimura, H., Setoyama, M. and Kato, H. (1980) Gallstones in Western Japan. Factors affecting the prevalence of intrahepatic gallstones. *Gastroenterology*, **78**, 684–690.
12. Tabata, M. and Nakayama, F. (1981) Bacteria and gallstones. Etiological significance. *Dig. Dis. Sci.*, **26(3)**, 218–224.
13. Saharia, P. C., Zuidema, G. D. and Cameron, J. L. (1977) Primary common duct stones. *Ann. Surg.*, **185(5)**, 598–604.
14. Chang, T. and Passaro, E. (1983) Intrahepatic stones: The Taiwan Experience. *Am. J. Surg.*, **146**, 214–264.
15. Matsumoto, Y., Fujii, H., Yoshioka, M., Sekikawa, T., Wada, T., Yamamoto, M., Eguchi, H. and Sugahara, K. (1986) Biliary strictures as a cause of intrahepatic bile duct stones. *World J. Surg.*, **10**, 867–875.
16. Miyake, H. A. (1913) Statistische, klinische und chemische Studien zur Aetiologie der Gallensteine mit besonderer Berücksichtigung der Japanischen und Deutschen Verhältnisse. *Klinische Chirurgie*, **101**, 54–117.
17. Sato, T., Matsushiro, T. and Suzuki, T. (1977) Results of surgical treatment for intrahepatic gallstones. *Tohoku J. Exp. Med.*, **122**, 303–312.
18. Bove, P., Ramos de Oliveria, M. and Speramzini, M. (1963) Intrahepatic lithiasis. *Gastroenterology*, **44**, 251–256.
19. Matsushiro, T., Suzuki, N. and Sato, N. (1977) Effects of diet on glucoric acid concentration in bile and the formation of calcium bilirubinate gallstone. *Gastroenterology*, **72**, 630–633.
20. Keddie, N. C., Gough, A. L. and Galland, R. B. (1976) Acalculous gallbladder disease: A prospective study. *Brit. J. Surg.*, **63**, 797.
21. Glenn, F. and Becker, C. G. (1982) Acute acalculous cholecystitis. *Ann. Surg.*, **195(2)**, 131–136.
22. Glenn, F. (1979) Acute acalculous cholecystitis. *Ann. Surg.*, **189(4)**, 458–465.
23. Hermann, R. E. and Grundfest-Broniatowski, S. (1988) Chronic cholecystitis. In *Surg. liv. bil. tract*, edited by L.H. Blumgart, **1**, 541–559.
24. Saik, R. P., Greenburg, A. G., Farris, J. M. and Peskin, G. W. (1975) Spectrum of cholangitis. *Am. J. Surg.*, **130**, 1143–149.
25. Goldmamn, L. D., Steer, M. L. and Silen, W. (1983) Recurrent cholangitis after biliary surgery. *Am. J. Surg.*, **145**, 450.
26. Lam, S. K., Wong, K. P., Chan, P. K., Nagan, H. and Ong, G. B. (1978) Recurrent pyogenic cholangitis: a study by endoscopic retrograde cholangiography. *Gastroenterology*, **74**, 1196.
27. Hultburn, A., Jacobsson, B. and Rosengren, B. (1962) Cholangiovenous reflux during cholangiography. *Acta Chirurgica Scandinavia*, **123**, 111.
28. Ong, G. B. (1962) A study of recurrent pyogenic cholangitis. *Arch. Surg.*, **84**, 63–89.
29. Ong, G. B., Adiseshiah, M. and Leong, C. H. (1971) Acute pancreatitis associated with recurrent pyogenic cholangitis. *Brit. J. Surg.*, **58**, 89–894.
30. Lim, J. H. (1991) Oriental cholangitis: pathological, clinical and radiological features. *Am. J. Roentgen.*, 157, 1–8.
31. Carmona, R. H., Crass, R. A., Lim, R. C. and Turnkey, D. D. (1984) Oriental cholangitis. *Am. J. Surg.*, **148**, 117–124.
32. Ho, C. S. and Wesson, D. E. (1974) Recurrent pyogenic cholangitis in Chinese immigrants. *Am. J. Roenten.*, **122**, 368–374.
33. Evans, H., Bourgeois, C. S., Comar, D. S. and Keschamras, N. (1971) Biliary tract changes in opisthorchiasis. *Am. J. Trop. Med. Hyg.*, **20**, 667–671.
34. Wong, W. T., Bettelheim, K. A., Cheng, F. C. Y. and Ong, G. B. (1982) Serotypes of Escherichia coli isolated from patients with recurrent pyogenic cholangitis. *J. Hyg.*, **88**, 513.
35. Cook, J., Hou, P. C. and Ho, H. C. (1954) Recurrent pyogenic cholangitis. *Brit. J. Surg.*, **42**, 188–203.
36. Wilde, H. (1973) Biliary calculus associated opisthorchiasis. *Am. J. Trop. Med. Hyg.*, **6**, 819–820.
37. Digby, K. H. (1930) Common-duct stones of liver origin. *Brit. J. Surg.*, **17**, 578–591.
38. Stock, F. E. and Trinkler, L. F. (1955) Choledochoduodenostomy in the treatment of cholangiohepatitis. *Surg., Gynec. Obstet.*, **101**, 599–606.
39. Federle, M. P., Cello, J. P., Laing, F. C. and Jeffrey, R. B. (1982) Recurrent pyogenic cholangitis in Asian immigrants. *Radiology*, **143**, 151.
40. Fung, J. (1964) Liver fluke infestation and cholangio-hepatitis. *Brit. J. Surg.*, **48**, 404–415.
41. Harrison-Levy, A. (1962) The biliary obstruction syndrome of the Chinese. *Brit. J. Surg.*, **49**, 674–685.
42. Menu, Y., Lorphelin, J.-M., Scherrer, A., Grenier, P. and Nahum, H. (1985) Sonographic and computed tomographic evaluation of intrahepatic calculi. *Am. J. Roentgen.*, **145**, 579–583.
43. Schulman A. (1987) Non-Western pattern of biliary stones and the role of ascariasis. *Radiology*, **162**, 425–430.
44. Turner, W. W. and Cramer, C. R. (1983) Recurrent oriental cholangiohepatitis. *Surgery* **93**, 397.

45. Afshani, P., Littenberg, G. D., Wollman, J. and Kaplowitz, N. (1978) Significance of microscopic cholangitis in alcoholic liver disease. *Gastroenterology*, **75**, 1045.
46. Ishak, K. G. and Rogers, W. A. (1981) Cryptogenic acute cholangitis: Association with toxic shock syndrome. *Am. J. Chest Phys.*, **75**, 619.
47. Ludwig, J., LaRusso, N. F. and Wiesner, R. H. (1990) The syndrome of primary sclerosing cholangitis. *Progr. Liver Dis.*, **9**, 555–565.
48. Meyers, R. N., Cooper, J. H. and Padis, N. (1970) Primary sclerosing cholangitis. *Am. J. Gastroenter.*, **53**, 527–538.
49. Wiesner, R. H. and LaRusso, N. F. (1980) Clinicopathologic features of the syndrome of primary sclerosing cholangitis. *Gastroenterology*, **79**, 200–206.
50. Warren, K. W., Athanassiades, S. and Monge, J. I. (1966) Primary sclerosing cholangitis: A study of forty-two cases. *Am. J. Surg.*, **111**, 23.
51. Chapman, R. W., Varghese, Z., Gaul, R., Patel, G., Kakinou, N. and Sherlock, S. (1983) Association of primary sclerosing cholangitis with HLA-B8. *Gut*, **24**, 38–41.
52. Andrews, E. and Henry, L. D. (1935) Bacteriology of normal and diseased gallbladders. *Arch. Int. Med.*, **56**, 1171–1188.
53. Edlund, Y. A., Mollstedt, B. O. and Ouchterlony, O. (1958) Bacteriological investigation of the biliary system and liver in biliary tract disease correlated to clinical data and microstructure of the gallbladder and liver. *Acta Chirurgica Scandinavica* **116**, 461–476.
54. Mason, G. R. (1968) Bacteriology and antibiotic selection in biliary tract surgery. *Arch. Surg.*, **97**, 533–537.
55. Csendes, A., Fernandez, M. and Uribe, P. (1975) Bacteriology of the gallbladder bile in normal subjects. *Am. J. Surg.*, **129**, 629, 1975.
56. Keighley, M. R. B. (1977) Micro-organisms in the bile. *Ann. Roy. Coll. Surg. Eng.*, **59**, 329–334.
57. Jackman, F. R., Hilson, G. R. F. and Lord Smith of Marlow (1980) Bile bacteria in patients with benign bile duct stricture. *Brit. J. Surg.*, **67**, 329–332.
58. Martin, C. F., Zinner, S. H., Kogan, J. P., Zametkin, A. J., Garrity, F. L. and Fry, D. E. (1983) Bacteriology of the human gallbladder in cholelithiasis and cholecystitis. *Am. Surg.*, **49**, 151–154.
59. Truedson, H., Elmros, T. and Holm, S. (1983) The incidence of bacteria in gallbladder bile at acute and elective cholecystectomy. *Acta Chirurgica Scandinavica*, **149**, 307–313.
60. Wells, G. P., Taylor, E. W., Lindsay, G. and Moston, L. (1989) Relationship between bile colonization, high-risk factors and postoperative sepsis in patients undergoing biliary tract operations while receiving a prophylactic antibiotic. *Brit. J. Surg.*, **76**, 374–377.
61. Anderson, R. E. and Priestly, J. T. (1951) Observations on the Bacteriology of Choledochal Bile. *Ann. Sur.*, **133**, 486.
62. Flemma, R., Flint, L. M. and Osterhout, S. (1967) Bacteriologic studies of biliary tract infection, *Ann. Surg.*, **166**, 563–572.
63. Scott, A. J. and Kahn, G. A. (1967) Origin of bacteria in bile duct bile. *Lancet*, **2**, 790–792.
64. Goswitz, J. T. (1974) Bacteria and biliary tract disease. *Am. J. Surg.*, **128**, 644–646.
65. Lygidakis, N. J. (1982) Incidence of bile infection in patients with choledocholithiasis. *Am. J. Gastroenter.*, **77**, 12–17.
66. Gregg, J. A., Girolami, P. D. and Carr-Locke, D. L. (1985) Effects of sphincteroplasty and endoscopic sphincterotomy on the bacteriologic characteristics of the common bile duct. *Am. J. Surg.*, **149**, 668–671.
67. Allen, J. I., Allen, M. O., Olson, M. M., Gerding, D. N., Shanholtzer, C. J., Meier, P. B., Vennes, J. A. and Silvis, S. E. (1987) Pseudomonas infection of the biliary system resulting from use of a contaminated endoscope. *Gastroenterology*, **92**, 759–763.
68. Elliot, D. W. (1980) Prevention of sepsis in biliary surgery. In *Controversies in surgical sepsis.* edited by S. Karran, pp. 285–291, Praeger, Sussex.
69. Benchimol, D., Bernard, J. L., Mouroux, J., Dumas, R., Elkaim, D., Chazal, M., Bourgeon, A. and Richelme, H. (1992) Infectious complications of endoscopic retrograde cholangiopancreatography managed in a surgical unit. *Inter. Surg.*. **77**, 270–273.
70. Lotveit, T. (1983) Bacterial infections of the liver and biliary tract. *Scandinavian Journal of Gastroenterology*. Supplement, **85**, 33–36.
71. Sung, J. Y., J. Y., Leung, J. W. C., Shaffer, E. A., Lam, K., Olson, M. E. and Costerton, J. W. (1992) Ascending infection of the biliary tract after surgical sphincterotomy and biliary stenting. *Journal of Gastroenterology and Hepatology*, **7(3)**, 240–245.
72. Escourrou, J., Cordova, J. A., Lazorthes, F., Frexinos, J. and Ribet, A. (1984) Early and late complications after endoscopic sphinterotomy for biliary lithiasis with and without the gallbladder 'in situ'. *Gut*, **25**, 598–602.
73. Neoptolemos, J. P., Davidson, B. R., Shaw, D. E., Carr-Locke, D. L. and Fossard, D. P. (1987) Study of common bile duct exploration and endoscopic sphincterotomy in a consecutive series of 438 patients. *Brit. J. Surg.*, **74**, 916–921.
74. Vaira, D., Ainley, C. and Williams, S. (1989) Endoscopic sphincterotomy in 1000 consecutive patients. *Lancet*, 431–434.
75. Dinen, P. (1964) The importance of the route of infection in experimental biliary tract obstruction. *Surg., Gynec. Obstet.*, **119**, 1001–1008.
76. Sung, J. Y., Shaffer, E. A., Olson, M. E., Leung, J. W. C., Lam, K. and Costerton, J. W. (1991) Bacterial invasion of the biliary system by way of the portal-venous system. *Hepatology*, **14**, 313–317.
77. Orloff, M. J., Peskin, G. W. and Ellis, H. L. (1958) A bacteriologic study of human portal bloods: implications regarding hepatic ischemia in man. *Ann. Surg.*, **148**, 738–746.
78. Schatten, W. E., Desprez, J. D. and Holden, W. D. (1955) Bacteriologic study of portal vein blood in man. *Arch. Surg.*, **71**, 404–409.
79. Straberg, S. M., Dorn, B. C., Small, D. M. and Egdahl, R. H. (1971) The effect of biliary tract pressure on bile flow, bile salt secretion and bile salt synthesis in the primate. *Surgery*, **70**, 140.
80. Stewart, L., Pellegrini, C. A. and Way, L. W. (1986) Antibacterial activity of the bile acids against common biliary tract organisms. *Surgical Forum*, **37**, 157.
81. Laskin, D. L. (1990) Nonparenchymal cells and hepatotoxicity. *Semin Liver Dis*, **10**, 293.
82. Fubara, E. S. and Freter, R. (1973) Protection against enteric bacterial infection by secretory IgA antibodies. *J. Immun.*, **111**, 395.

